# Novel Granulectomy Technique and Intralesional Triamcinolone for Liquid Injectable Silicone-Induced Chin Granulomas: A Long-Term Ambidirectional Cohort Study

**DOI:** 10.3390/jcm15176506

**Published:** 2026-08-22

**Authors:** Ago Harlim, Hubert Andrew, Chastine Harlim

**Affiliations:** 1 Department of Dermatology and Venereology, Faculty of Medicine, Universitas Kristen Indonesia, Jakarta 13630, Indonesia; 2 Faculty of Medicine, Universitas Indonesia, Jakarta 10430, Indonesia; hubertandrewlimm@gmail.com (H.A.); drchastineharlim@gmail.com (C.H.)

**Keywords:** silicone granuloma, foreign body reaction, granulectomy, intralesional steroids, liquid injectable silicone

## Abstract

**Background**: Liquid injectable silicone (LIS) is controversially used for soft-tissue augmentation, frequently causing severe, late-onset foreign body granulomas. Complete surgical removal is nearly impossible due to silicone migration, with residues leading to high recurrence. Meanwhile, medical therapies alone do not address macroscopic structural deformity. This study evaluates a combined protocol of subdermal granulectomy followed by adjuvant intralesional steroids for chin siliconomas. **Methods**: An ambidirectional cohort study (2015–2025) evaluated 35 patients with LIS-induced chin granulomas. All subjects underwent submental granulectomy. Postoperatively, patients were allocated to an adjuvant steroid cohort (*n* = 17) or a surgery-only control cohort (*n* = 18). The outcomes included aesthetic improvements (chin shape and erythema) and recurrence-free survival. **Results**: Granulectomy yielded significant improvements in aesthetics (*p* = 0.001) and erythema (*p* < 0.001) with a 2.86% complication rate. Longitudinal survival analysis demonstrated that adjuvant steroids lowered the crude recurrence rate (5.89% vs. 44.4%, *p* = 0.018) and significantly extended recurrence-free survival (Log-rank *p* = 0.016). The surgery-only cohort exhibited higher recurrence risk (Cox hazard ratio = 8.59, *p* = 0.043). Over the follow-up period, the surgery-only cohort experienced significant aesthetic deterioration (*p* = 0.04) and persistent redness, whereas the steroid cohort preserved postsurgical chin shape (*p* = 0.19) and achieved further erythema reduction (*p* = 0.03). Multivariable regression indicated higher odds of aesthetic preservation (adjusted odds ratio [aOR] = 3.85, *p* = 0.082) and erythema resolution (aOR = 2.90, *p* = 0.145) in the steroid cohort, although statistical significance was not reached. **Conclusions**: Our subdermal granulectomy technique successfully removed macroscopic silicone burden while maintaining the appearance of the anterior chin. The implementation of an early adjuvant steroid protocol provides a synergistic advantage by neutralizing residual microscopic inflammation, delaying recurrence, and ensuring durable aesthetic outcomes.

## 1. Introduction

The desire for aesthetic enhancement has led to a global rise in the use of injectable fillers on various parts of the body, including the chin. Among the substances used, liquid injectable silicone (LIS) is controversially used off-label for soft-tissue augmentation, leading to a high rate of severe, and rarely, fatal complications [1]. One of the most common complications of LIS injection or other fillers is the formation of foreign body reactions (granuloma), characterized by various grades of chronic inflammation and fibrosis [2]. These granulomas can cause significant cosmetic disfigurement, pain, and psychological distress, often appearing years after the initial injection [1,3].

The management of LIS granulomas is notoriously challenging due to the migratory nature of silicone microdroplets, which can infiltrate surrounding tissues, making complete surgical removal nearly impossible. No consensus currently exists on the treatment of foreign-body granuloma. Contemporary modalities include surgical excision, systemic and intralesional corticosteroids, antibiotics, and immunomodulatory agents, with varying degrees of success and a high rate of recurrence [1,4]. Surgical evacuation (curettage or granulectomy) as a standalone procedure leaves microscopic silicone residue, leading to scarring, post-operative contour irregularities, and high rates of recurrence and symptom flare-ups [5]. On the other hand, intralesional corticosteroids (such as triamcinolone acetonide) may be effective in reducing acute inflammation and fibrosis, but its use alone fails to address the underlying structural deformity [6]. Thus, a multimodal approach is necessary in the treatment of LIS granulomas.

Through an ambidirectional cohort analysis spanning 10 years, this study evaluates the long-term recurrence rate and aesthetic outcomes of a novel granulectomy technique in combination with intralesional steroid injections for LIS-induced granuloma.

## 2. Materials and Methods

### 2.1. Study Design

An ambidirectional observational cohort study was conducted to obtain both retrospective and prospective data via total sampling from patients who underwent excision of LIS-induced granuloma of the chin between 2015 and 2025 in our clinic. To evaluate the efficacy of postoperative intralesional steroid injections, the study population was subsequently split into two cohorts: a surgery-only cohort and a steroid cohort.

All enrolled patients initially underwent a standardized granulectomy with a novel technique (performed as described in the next section). Granulectomy assessment was based on the last surgery. Two weeks after removal of the granulectomy sutures, treatment allocation was determined by patient preference following comprehensive counseling. During the postoperative consultation, all patients were offered adjuvant steroid injections and informed of the potential risks and benefits. Patients who declined the adjuvant therapy generally did so due to fear of injection-site pain or logistical constraints regarding repeated visits. Those who consented to steroid injections were allocated to the steroid cohort and received adjuvant intralesional triamcinolone acetonide (10 mg/mL) injections administered at 0.1–0.2 mL/cm^2^ [7]. The protocol targeted at least a total of three to seven injections given at 1–2-week intervals, titrated to individual clinical response. Meanwhile, the comparison cohort received surgery only without adjuvant steroid injections. The steroid protocol can be found in Supplementary Materials File S1.

Throughout the study, incidence of recurrence and surgical complications (bleeding, infection, persistent swelling of >2 weeks, wide seroma, and skin perforation) were recorded.

Finally, all patients were contacted from January to February 2026 to return to the clinic for a final follow-up to evaluate long-term aesthetic outcomes of their respective surgery. Those who were unable to come to the clinic were asked to provide photos and their subjective perception of current results using an online questionnaire.

### 2.2. Eligibility Criteria

The inclusion criteria in this study were (1) adult patients clinically diagnosed with LIS-induced granuloma localized to the chin region; (2) a history of granulectomy performed at our clinic during study timeframe; (3) available photographic documentation across all time points. Those with a non-LIS etiology of granuloma, such as other injectables or infection, and those with concurrent long-term systemic immunosuppressive therapy (including corticosteroids and chemotherapy) during the follow-up period were also excluded.

### 2.3. Surgical Technique

**Figure 1:**
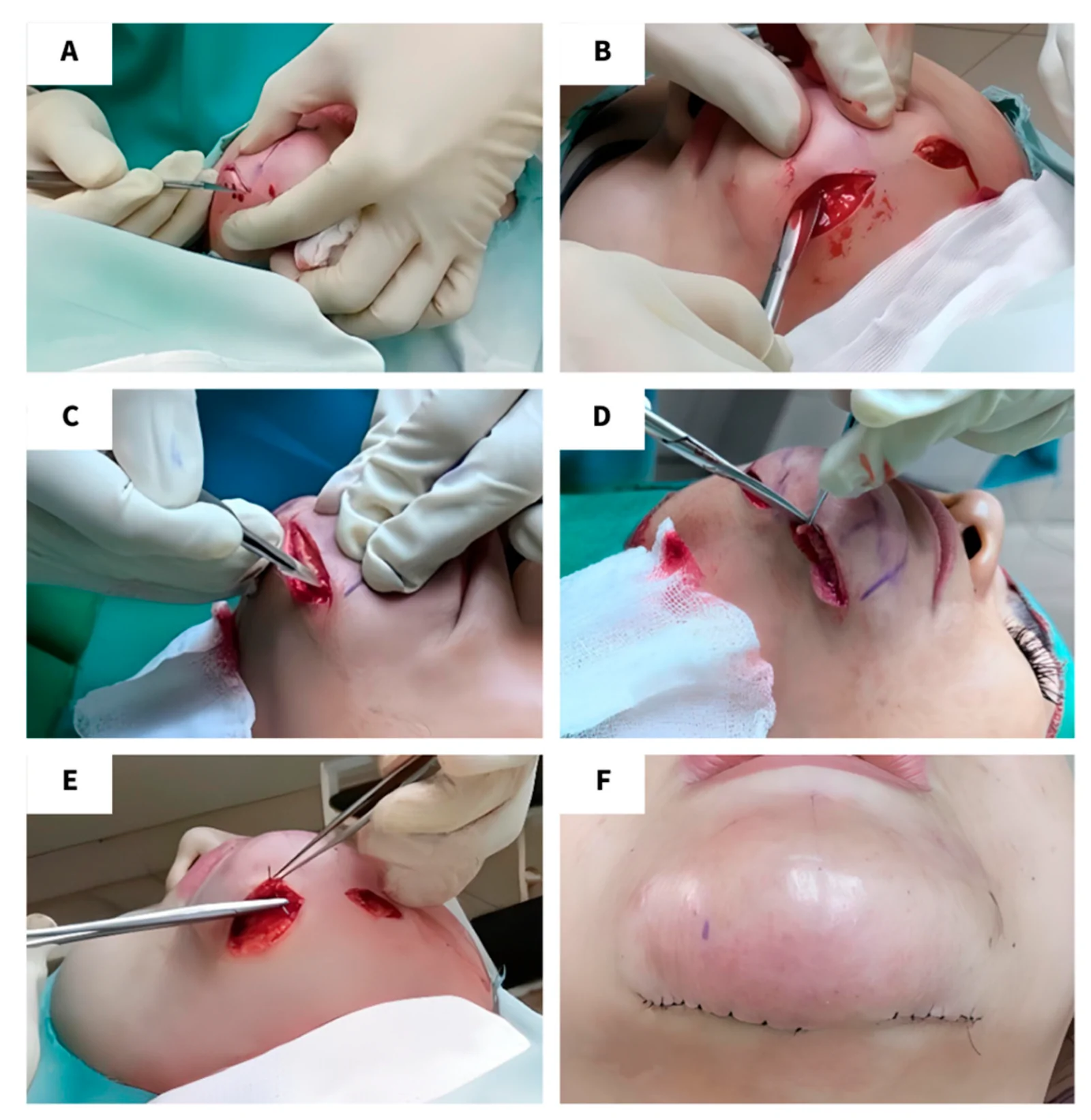
Surgical technique. (**A**) Skin incision on a previously locally anesthetized delineated excision area just inferior to the submental crease. (**B**) Palpation of the granuloma by undermining. (**C**) Eversion of the cutaneous flap to provide direct visualization while excising the granuloma from the bottom upward. Meticulous execution is necessary to prevent accidental damage to the skin above. (**D**) Extraction of the granuloma followed with blunt dissection of fibrotic bands and curettage of adherent remnants. (**E**,**F**) Closure of the incision with subcuticular skin closure.

The granulectomy technique we developed was modified from a mentoplasty procedure described by Willet (Figure 1) [8]. Following preoperative marking of the median line, granuloma, and topographic irregularities with gentian violet, the surgical field was sterilized with 10% povidone–iodine and 70% alcohol followed by local anesthesia with 2% lidocaine. An elliptical incision (3–5 cm) was made parallel to the submental line and undermining was performed beneath the granuloma. The cutaneous flap was then everted to provide direct visual access to the undersurface of the dermis, allowing the granuloma to be excised from the bottom upward using a No. 11 blade while strictly avoiding penetration of the overlying skin. Surgical scissors were used to dissect remaining fibrotic tissue and refine the excision cavity. Adherent remnants were treated with curettage and bleeding was controlled with cauterization. The sutures used were 4-0 Vicryl for soft tissue and 6-0 Prolene for subcuticular skin closure. The subcuticular stitches were removed one week after the procedure.

### 2.4. Ethics

The study was conducted in accordance with the Declaration of Helsinki. Informed consent was obtained from the respondents for inclusion in the research and publication, including medical record data and clinical photographs at all three timepoints. Ethical clearance for this ambidirectional study protocol was obtained from the Ethics Review Committee of Universitas Kristen Indonesia (Approval number: No. 0lA/Etik Penelitian/FKUKI/2019).

### 2.5. Data Analysis

Baseline characteristics were analyzed with descriptive statistics. Comparative statistics were performed to ensure baseline characteristics were comparable between the group who received steroids and those who did not. Numeric data were expressed as mean and standard deviation or median and minimum–maximum values, depending on normality. Ordinal and nominal data were expressed in frequency and percentage.

Kaplan–Meier survival analysis and Cox proportional hazards regression analysis were conducted to study the effect of steroid injections on the probability of recurrence over time and to estimate the hazard ratio of recurrence between the two groups. Paired comparison tests were conducted, namely paired T-tests and Wilcoxon signed-rank tests. Paired comparison tests comparing chin aesthetics and erythema were conducted twice, (1) before and after the surgery, to analyze the efficacy of the surgery, and (2) after the surgery and at final follow, to analyze the effect of steroid administration on granuloma regression between subjects who received the injections and those who did not. Multivariable analysis was performed to assess the effect of steroid administration on chin shape and erythema at follow-up. Data were analyzed using IBM SPSS Statistics version 26. A *p*-value of <0.05 was considered significant for all the statistical tests.

### 2.6. Data Collection and Outcomes

Data were collected both retrospectively from medical records (demographics and operative details) and prospectively during follow-up. Patients who returned to our clinic presenting with signs or symptoms at the site previously operated on were recorded as recurrent cases. The longitudinal outcome for the survival analysis was the number of recurrence-free weeks, which was the number of weeks from the date of the granulectomy to either the recurrence of the granuloma or the date of the final follow-up (censored data).

Aesthetic outcomes (chin shape and erythema severity) were evaluated with an ordinal grading scale (Table 1, see Supplementary Materials File S1 for the photographic assessment protocol). Standardized clinical photographs (anterior, bilateral profiles, and bilateral obliques) were assessed at three discrete timepoints: (1) baseline pre-operative (immediately before the procedure), (2) baseline post-operative (one-week post-granulectomy), and (3) final follow-up. The pre-operative and immediate post-operative photographs were graded exclusively by an independent, blinded physician evaluator. Conversely, to capture both clinical outcomes and patient satisfaction, the aesthetic grade at the final follow-up was calculated as the average between the patient’s subjective self-assessment and the blinded physician’s objective evaluation [9]. In cases where the mathematical average of the physician’s evaluation and the patient’s self-reported score did not result in a whole number, a senior physician acted as a blinded tiebreaker to adjudicate the final integer.

## 3. Results

**Figure 2:**
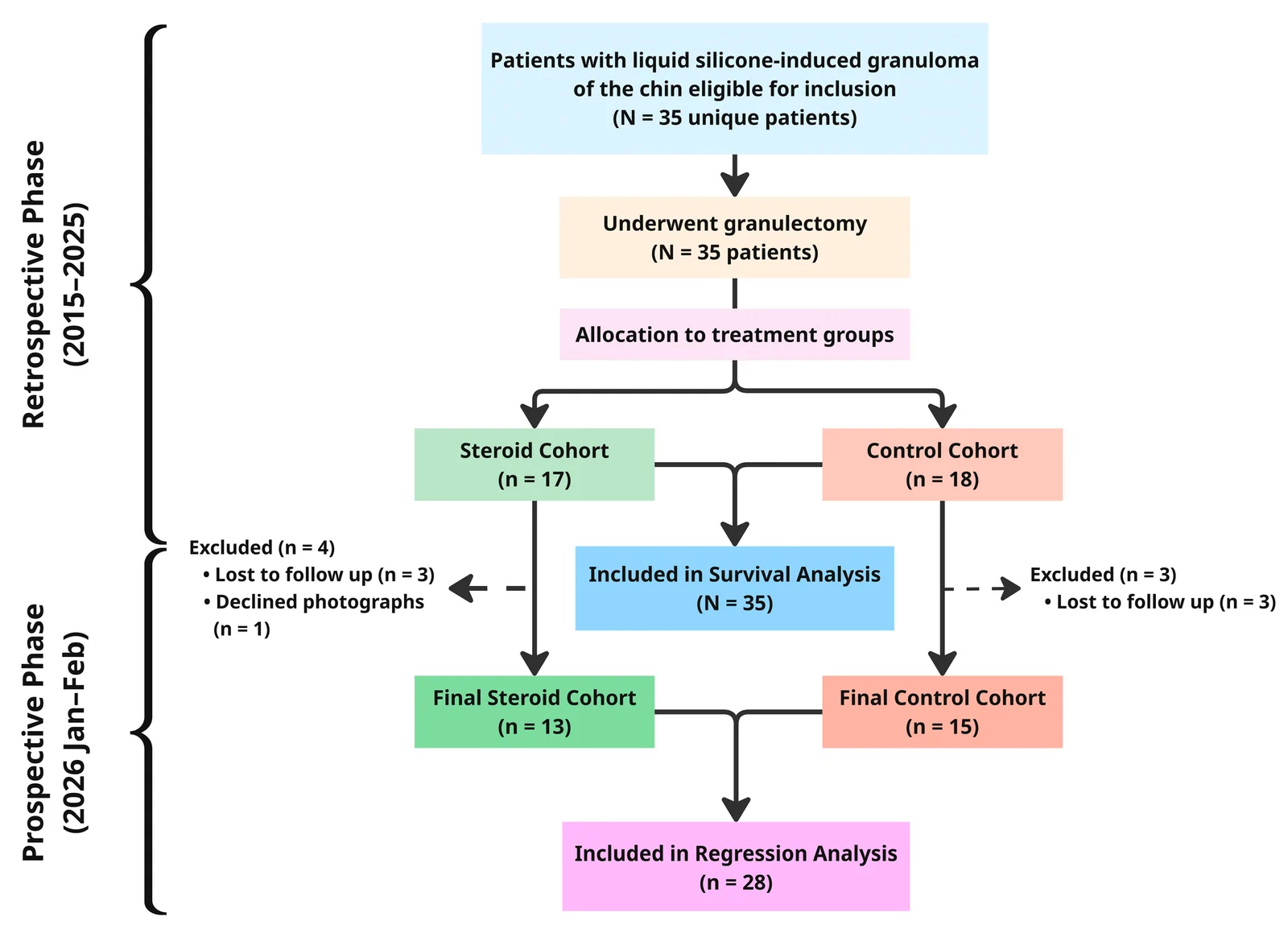
Study flowchart of the ambidirectional cohort design outlining patient progression across retrospective and prospective phases. The diagram encompasses enrollment, allocation, Kaplan–Meier and Cox survival analyses, attrition, and final evaluation of long-term aesthetic outcomes.

A total of 35 patients with LIS-induced chin granulomas underwent granulectomy in our clinic (Figure 2). Based on consent for steroid injections, they were then allocated into a primary steroid cohort (*n* = 17) and a primary surgery-only cohort (*n* = 18). At the prospective final follow-up (January–February 2026), 7 patients were excluded from the final aesthetic analysis. In the steroid cohort (*n* = 17), 4 patients were missing (3 were lost to follow-up; 1 declined to provide clinical photographs). In the surgery-only cohort (*n* = 18), 3 patients were lost to follow-up. Consequently, the final multivariable ordinal regression for aesthetic outcomes was conducted on the remaining 28 patients.

### 3.1. Baseline Characteristics

To ensure the validity of subsequent analyses, the primary cohorts were analyzed for homogeneity (Table 2). There were no statistically significant differences between the two cohorts regarding age (51.41 ± 7.87 vs. 48.56 ± 11.53; *p* = 0.401) or sex distribution (*p* = 0.486). Furthermore, there were no statistically meaningful differences in baseline pre-operative chin aesthetics and erythema intensity between the cohorts (*p* > 0.05). Importantly, following granulectomy, both cohorts had similar post-operative baseline for both chin shape and redness (*p* > 0.05), verifying that any subsequent differences in outcomes were not confounded by initial chin deformity or surgical intervention.

### 3.2. Clinical Outcomes

#### 3.2.1. Evaluation of Surgical Efficacy

**Figure 3:**
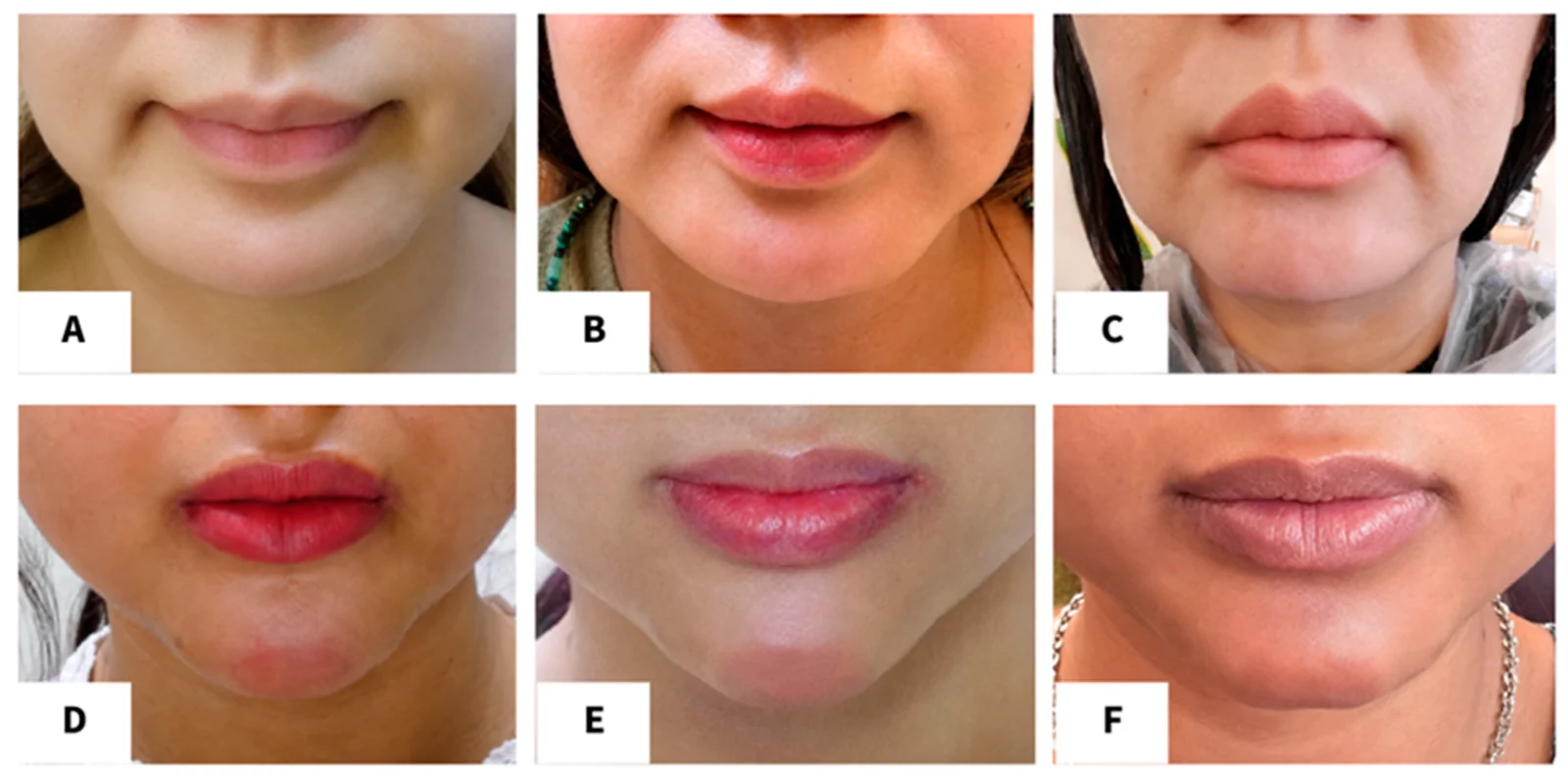
Representative clinical case outcomes for the surgery-only and steroid cohorts. From left to right, the columns represent the three points in data collection: (1) pre-operative baseline presentation, (2) one-week after the surgery, and (3) final follow-up. Panels (**A**–**C**): longitudinal sequence for a subject from the surgery-only cohort. (**C**) Final follow-up at 159 weeks after the surgery. Panels (**D**–**F**): longitudinal sequence for a subject from the steroid cohort. (**F**) Final follow-up after five adjuvant steroid injections. The picture was taken 125 weeks after the surgery.

To assess the immediate clinical efficacy of the granulectomy technique (Figure 3, Columns 1–2), a Wilcoxon signed-rank test was conducted comparing baseline pre-operative and post-operative scores (*N* = 35). The test demonstrated significantly improved chin aesthetics following surgery (Z = −3.240, *p* = 0.001). Similarly, erythema scores significantly decreased after the granulectomy (Z = −4.191, *p* < 0.001), denoting an amelioration in skin redness (Table 3).

The granulectomy procedure was well-tolerated across all 35 cases, with no major surgical complications. One case developed postoperative seroma, which spontaneously resolved within one week without intervention, yielding an overall complication rate of 2.86% (*n* = 1). Notably, postoperative swelling resolved within one week in all cases. No skin perforations occurred during the procedure and all incisions healed favorably.

#### 3.2.2. Long-Term Aesthetic and Erythema Outcomes

To evaluate granuloma progression following surgery in each cohort (Figure 3, Column 3), an intra-group Wilcoxon signed-rank test was performed comparing the post-operative scores to the final follow-up scores (Table 4). In the surgery-only cohort (*n* = 15), post-surgical chin shape significantly deteriorated over the follow-up period (Z = −2.02, *p* = 0.043), while erythema scores showed no significant resolution (Z = −0.60, *p* = 0.55). On the other hand, the steroid cohort demonstrated preservation of their post-operative chin aesthetics with no significant degradation in shape (Z = −1.31, *p* = 0.19), alongside a statistically significant improvement in erythema (Z = −2.16, *p* = 0.03).

Ordinal logistic regression was conducted to determine the effect of post-operative steroid injections on both chin shape and erythema at follow up. The analysis adjusted for two confounders, namely baseline postoperative scores and time to final assessment (Table 5). The model suggests that adjunctive steroid injections yield a substantial clinical effect size, though it did not reach statistical significance, which was likely due to the limited sample size.

Cases in the injection cohort had nearly four times the adjusted odds of achieving superior chin shape at follow up (aOR = 3.85; 95% CI: 0.83–17.74, *p* = 0.082) and almost three times the odds of achieving a better erythema resolution (aOR = 2.90; 95%CI: 0.69–12.12, *p* = 0.145) compared to the surgery-only cohort. Furthermore, neither baseline post-operative scores nor the time to final assessment significantly influenced either outcome, indicating steroid administration as the primary factor of clinical outcome. The complete SPSS outputs can be found in the Supplementary Materials File S1.

#### 3.2.3. Survival Analysis and Recurrence of Chin Granulomas

**Figure 4:**
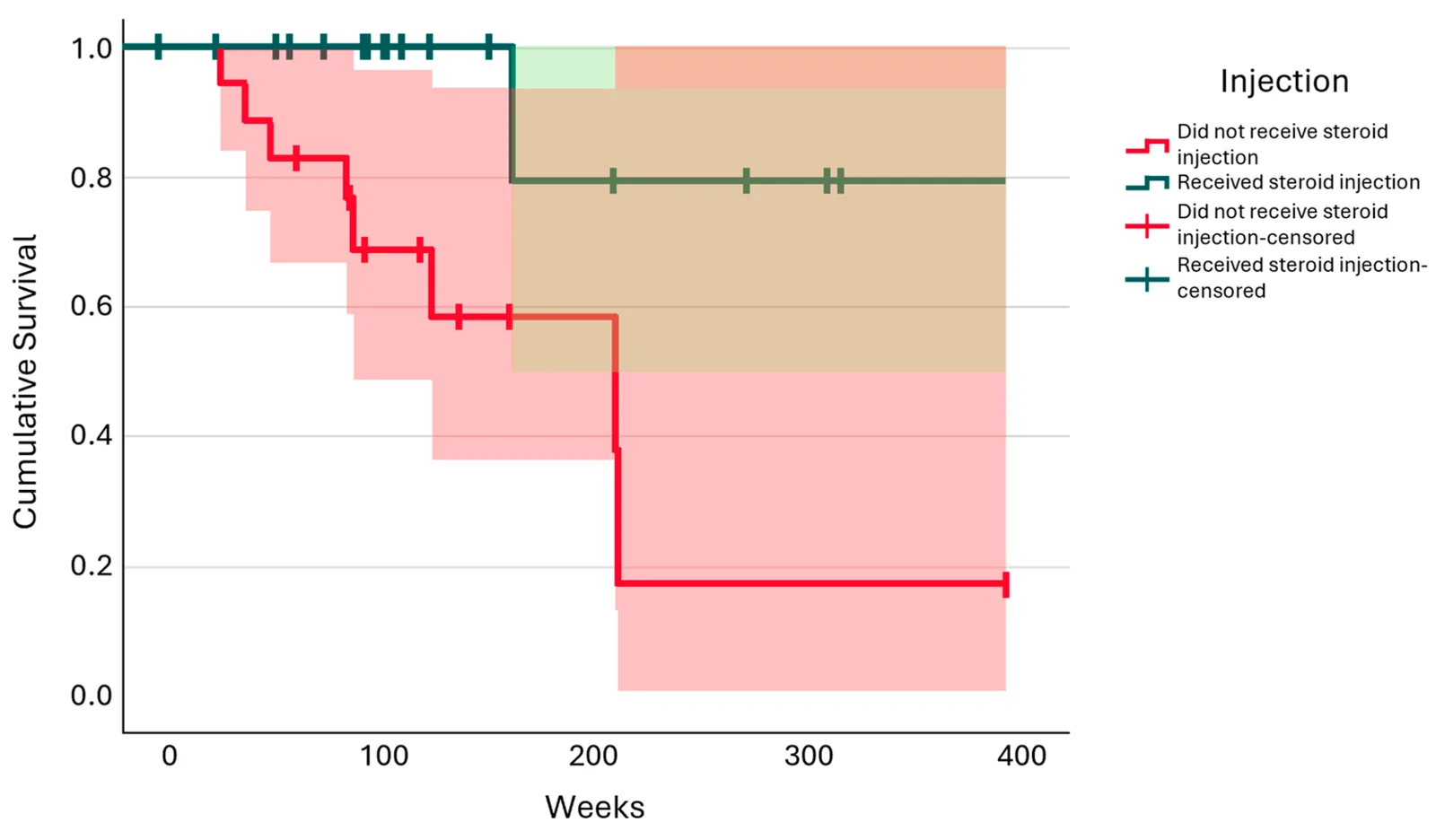
Kaplan–Meier curves illustrating cumulative recurrence-free survival of the steroid and control groups along with their respective 95% confidence intervals. The steroid cohort exhibited a significantly higher cumulative recurrence-free time (Log-rank test, χ^2^ = 5.85, *p* = 0.016). Cox regression analysis indicated that the surgery-only group possessed an 8.59-fold increased hazard of granuloma recurrence compared to the steroid group (95% CI: 1.07–69.13; *p* = 0.043).

For the survival cohort (*N* = 35), the mean follow-up duration was 137.85 weeks (range: 16–385 weeks). The crude recurrence rate was significantly lower in the steroid injection cohort (5.89%, *n* = 1/17) compared to the surgery-only cohort (44.4%, *n* = 8/18; *p* = 0.018, Fisher’s exact test). A subsequent Kaplan–Meier survival analysis demonstrated a significant improvement in recurrence-free survival time for patients receiving steroid injections (Log-rank test, χ^2^ = 5.85, *p* = 0.016; Figure 4). The median recurrence-free survival for the surgery-only cohort was 215 weeks (95% CI: 68.51–361.49), while the median was not reached for the steroid cohort during the follow-up period. Further evaluation with Cox proportional hazards regression revealed that patients in the surgery-only cohort had a significantly higher risk of recurrence compared to those receiving adjuvant steroids (hazard ratio = 8.59; 95% CI: 1.07–69.13; *p* = 0.043).

## 4. Discussion

The risks associated with LIS injections have long been identified, including immune responses, migration, tissue discoloration, and granuloma formation [10]. While granuloma formation is not unique to silicone injections, the management of silicone-induced chin granuloma represents a distinct challenge. Although silicone is chemically inert and nontoxic, its hydrophobic property triggers the Vroman effect, i.e., the adsorption of plasma protein on its surface. This newly formed protein matrix triggers an immune response resulting in clotting, fibrinolysis, and subsequent activation of the inflammatory cascade and recruitment of phagocytic cells such as macrophages [2].

Injected fillers form into microdroplets measuring 20–100 μm in size. Smaller microdroplets (<15 μm) are phagocytosed and cleared through lymphatic vessels. However, larger microdroplets, which are indigestible due to their size and non-biodegradability, like LIS, will cause macrophages to undergo “frustrated phagocytosis”. These cells will fuse into giant cells adhering to the surface of the foreign material, producing a microenvironment rich in degradation mediators, such as reactive oxygen species, enzymes, and acids, while also recruiting other immune cells such as lymphocytes. The chronic and persistent inflammation, mediated by T-helper cells, eventually results in the formation of fibrotic capsules, which is mainly driven by tumor necrosis factor (TNF)-α along with interleukin (IL)-6 and IL-13. Furthermore, due to the droplets being liquid, they may migrate along tissue planes, causing widespread tissue damage. It is suggested that immune response to a foreign body is affected by individual immune tolerance [2,11,12].

Histologically, granulomas are characterized by the presence of multinucleated giant cells, macrophages, and lymphocytic or neutrophilic infiltration. Notably, these giant cells contain clear vacuoles of varying that correspond to the LIS or other fillers that have dissolved during histologic processing, resulting in a “Swiss cheese-like” appearance. Harlim et al. had previously proposed a histopathological grading of silicone granulomas, with seven categories mapping the transition from acute inflammation to end-stage tissue fibrosis [2].

Considering the pathophysiology of granuloma, which revolves around chronic inflammation, one can expect that early removal of an LIS granuloma would result in better aesthetic outcomes. However, complete surgical removal of a granuloma is often impossible, mainly due to the migratory and invasive nature of the LIS droplets, which leaves residual silicone and causes recurrence [4]. As such, our protocol involves a combined approach: subdermal granulectomy followed by a course of intralesional steroid injections. This surgical technique proved effective, demonstrating a significant improvement in chin aesthetics (*p* = 0.001) and erythema (*p* < 0.001) one week after the procedure with a low complication rate (2.86%).

As inflammation is a central process in the formation of granuloma, both local and systemic steroids serve an important role in its management. Steroid injections reduce fibroblast proliferation and collagen synthesis, inhibit macrophage activity, and decrease erythema [1,4]. Subjects in this study were administered at least three doses of intralesional 10 mg/mL triamcinolone every 1–2 weeks with a target of seven injections in total. The results of our study showed that post-granulectomy subjects receiving intralesional steroids experienced a longer duration without recurrence (*p* = 0.003). This suggests that steroid use may not completely prevent recurrence but does have a protective effect, i.e., delaying recurrence and prolonging recurrence-free duration. In our experience, steroid injections can also be used to address post-surgical irregularities, particularly in the lateral chin area, to suppress residual inflammation and fibrosis. The efficacy of this combined modality is highlighted by a subject who experienced recurrence at 216 weeks as a surgery-only case. After reoperation and reenrollment into the steroid cohort, the subject remained recurrence-free until the end of the study period (276 weeks).

Other mainstay pharmacological management techniques include tetracycline antibiotics (which show immunomodulatory activity towards inflammatory nodules, granulomas, and sterile abscesses) and non-steroidal anti-inflammatory drugs (which reduce granulomatous inflammatory reaction) along with novel therapies such as imiquimod (showing antiproliferative action), etanercept, adalimumab (a TNF-α inhibitor), tacrolimus (contributing to the inhibition of T-cell activation and release of mediators by mast cells), allopurinol (an xanthine oxidase inhibitor to prevent granuloma formation), and isotretinoin (which is anti-inflammatory) [1,4].

When contextualizing our low complication (2.86%) and recurrence (5.89%) rates against those in the existing literature, both anatomical burden and surgical methodology are critical. While massive gluteal en bloc resections inherently carry high risks of dehiscence and graft loss [5], anatomical localization alone does not explain our optimal outcomes. Even within the face, previously described management employs an open-facelift approach requiring superficial filler bursae to be left intact to prevent flap necrosis, or necessitates delayed fat grafting following the management of deep filler pockets [13].

In contrast, our protocol achieves granuloma removal and contour preservation in a single surgical stage. By utilizing a submental incision and everting the cutaneous flap, the dermal undersurface is visualized. Subsequent excision of the granuloma from the bottom upward allows for precise contour refinement and targeted hemostasis.

Furthermore, the long-term stability of the results can be attributed to our steroid protocol. Because complete surgical eradication of migrated liquid silicone is clinically impossible [4], our adjuvant protocol is essential, yielding a lower recurrence rate than surgery alone (5.89% vs. 44.4%). This protocol successfully suppressed residual chronic inflammation [12] without inducing the severe localized skin atrophy, pigmentary changes, or telangiectasias associated with higher-dose steroid regimens.

Less invasive approaches have also been described in the literature. Bowles et al. also demonstrated improvement in granuloma symptoms in three cases after employing intralesional injection of a mixture containing 5-fluorouracil, dexamethasone, and triamcinolone along with an oral regimen of colchicine and naproxen [6]. Improved aesthetic outcomes, along with reduced erythema and telangiectasias, were observed after a follow-up period of 5–12 months. Zarei et al. utilized a novel and less invasive approach using dermabrasion to physically drain migrating LIS in the lower legs. After initially failing to respond to methylprednisolone, cyclosporine, and liposuction, the patient underwent extensive bilateral dermabrasion to evacuate the silicone followed by intravenous steroids. After exudation of the silicone-laden fluid, the authors reported improvement in ambulation, adequate pain control, and appropriate wound healing [14]. Unlike the aforementioned methods, our technique is more straightforward, requiring fewer treatment steps and allowing easier implementation in daily clinical practice while maintaining favorable clinical outcomes.

The findings of this study must be interpreted within the context of its methodological limitations. First, the non-randomized allocation introduces an inherent risk of selection bias by potentially funneling more compliant patients into the treatment arm. Mitigation was achieved by confirming baseline homogeneity between the cohorts and adjusting for baseline post-operative severity in our multivariable regression. Second, subject dropouts during the lengthy study period introduces a risk of attrition bias. Long-term surveillance of patients residing in distant cities was also challenging. It is also possible that satisfied patients were less motivated to return for final evaluation. Nevertheless, this loss to follow-up inherently affects data completeness. Furthermore, remote photographic documentation was declined in one case due to cultural sensitivities regarding cosmetic procedures. Third, the consequent small sample size decreases the statistical power of the study, though the effect size of the steroid intervention remained large enough to achieve clinical significance. Notably, the survival analysis retained robust statistical significance despite the cohort size.

Despite these limitations, the primary value of this study lies in its extensive 10-year observational span and the isolation of chin-specific LIS granulomas. The combined submental granulectomy and adjuvant steroid protocol also demonstrated a reproducible framework while maximizing immediate and long-term aesthetics. Future multi-center randomized controlled trials are warranted to further optimize the adjuvant steroid dosing.

## 5. Conclusions

This study reports the management and long-term outcomes of LIS granuloma isolated specifically to the chin region. We propose a highly reproducible framework consisting of granulectomy followed by an early adjuvant steroid protocol. By utilizing a submental incision and cutaneous eversion, the subdermal granulectomy achieves precise granuloma excision and contour preservation. The adjuvant steroid injections provide a crucial synergistic advantage and long-lasting suppression by targeting microscopic residual inflammation, halting fibrosis, and significantly delaying recurrence.

## Figures and Tables

**Table 1:** Evaluation metrics for chin aesthetic and erythema (adapted from Global Aesthetic Improvement Scale).

Scale	5-Tier Scale	Description	3-Tier Scale	Description
Chin aesthetic	5	Very good	4–5	Good
	4	Good		
	3	Fair	3	Fair
	2	Poor	1–2	Poor
	1	Very poor		
Erythema severity	4	Very red	3	Red
	3	Red		
	2	Slightly red	2	Slightly red
	1	Not red at all	1	Not red at all

**Table 2:** Patient demographic and clinical characteristics.

Characteristic	Overall (*N* = 35)	Steroid Cohort (*n* = 17)	Control Cohort (*n* = 18)	*p*-Value
Demographic Characteristics				
Age (years), mean ± SD	49.94 ± 9.88	51.41 ± 7.87	48.56 ± 11.53	0.401 ^a^
Sex, n (%)				
Female	34 (97.1)	16 (94.1)	18 (100)	0.486 ^b^
Male	1 (2.9)	1 (5.9)	0	
Clinical Characteristics				
Pre-Op chin score, median (min–max)	4.0 (1.0–4.0)	4.0 (1.0–4.0)	4.0 (1.0–4.0)	0.858 ^c^
Pre-Op chin score, (%)				
Very good	0	0	0	1.00 ^b^
Good	24 (68.6)	12 (70.6)	12 (66.7)	
Fair	0	0	0	
Poor	9 (25.7)	4 (23.5)	5 (27.8)	
Very Poor	2 (5.7)	1 (5.9)	1 (5.6)	
Pre-Op redness score, median (min–max)	4.0 (1.0–4.0)	4.0 (1.0–4.0)	4.0 (2.0–4.0)	0.757 ^c^
Pre-Op redness score, (%)				
Very red	22 (62.9)	11 (64.7)	11 (61.1)	0.337 ^b^
Red	9 (25.7)	5 (29.4)	4 (22.2)	
Slightly red	3 (8.6)	0	3 (16.7)	
Not red at all	1 (2.9)	1 (5.9)	0	
Post-Op chin score, median (min–max)	4.0 (2.0–5.0)	4.0 (3.0–5.0)	4.0 (2.0–5.0)	0.386 ^c^
Very good	13 (37.1)	8 (47.1)	5 (27.8)	0.234 ^b^
Good	16 (45.7)	6 (35.3)	10 (56.5)	
Fair	4 (11.4)	3 (17.6)	1 (5.6)	
Poor	2 (5.7)	0	2 (11.1)	
Very Poor	0	0	0	
Post-Op redness score, median (min–max)	2.0 (1.0–4.0)	2.0 (1.0–4.0)	2.5 (1.0–4.0)	0.832 ^c^
Post-Op redness score, (%)				
Very red	8 (22.9)	4 (23.5)	4 (22.2)	0.828 ^b^
Red	9 (25.7)	4 (23.5)	5 (27.8)	
Slightly red	12 (34.3)	7 (41.2)	5 (27.8)	
Not red at all	6 (17.1)	2 (11.8)	4 (22.2)	

SD: standard deviation. ^a^: T-test. ^b^: Fisher’s exact test. ^c^: Mann–Whitney U test.

**Table 3:** Intra-group comparison of preoperative and 1-week post-operative scores.

Overall Cohort (*N* = 35)	Pre-Op Median (Min–Max)	Post-Op Median (Min–Max)	Median Difference (95% CI)	*p*-Value
Chin Shape Score	4.0 (1.0–4.0)	4.0 (2.0–5.0)	1.0 (0.5–1.5)	0.001 ^a,^*
Erythema Score	4.0 (1.0–4.0)	2.0 (1.0–4.0)	−1.0 (−1.0 to −0.50)	<0.001 ^a,^*

95% CI—95% confidence interval. ^a^: Wilcoxon signed-rank test. *: Statistically significant.

**Table 4:** Intra-group comparison of 1-week post-operative scores and final follow-up scores.

**Steroid Cohort (*n* = 13)**	**Baseline Post-Op Median (Min–Max)**	**Final Median (Min–Max)**	**Median Difference (95% CI)**	***p*** **-Value**
Chin Shape Score	4.0 (3.0–5.0)	4.0 (2.0–5.0)	−0.5 (−1.0 to 0.5)	0.190 ^a^
Erythema Score	2.0 (1.0–4.0)	2.0 (1.0–3.0)	−0.5 (−1.5 to 0.0)	0.031 ^a,^*
**Control cohort (*n* = 15)**	**Baseline Post-Op Median (Min–Max)**	**Final Median (Min–Max)**	**Median Difference (95% CI)**	***p*** **-value**
Chin Shape Score	4.0 (2.0–5.0)	3.0 (2.0–5.0)	−1.0 (−1.5 to 0.0)	0.043 ^a,^*
Erythema Score	3.0 (1.0–4.0)	2.0 (1.0–4.0)	−0.25 (−1.0 to −0.50)	0.547 ^a^

95% CI—95% confidence interval. ^a^: Wilcoxon signed-rank test. *: Statistically significant.

**Table 5:** Chin and redness score before and after surgery, and at final follow-up.

Predictor Variable	Chin Shape at Follow-Up ^a^			Erythema at Follow-Up ^a^		
	aOR ^b^	95% CI	*p*-Value	aOR ^b^	95% CI	*p*-Value
**Intervention**						
No Steroid Injection	1.00 (Ref)	—	—	1.00 (Ref)	—	—
Steroid Injection	3.85	0.83–17.74	0.082	2.90	0.69–12.12	0.145
**Covariates**						
Baseline Post-Op Chin Shape Score	0.83	0.23–2.97	0.771	N/A		
Baseline Post-Op Erythema Score	N/A			0.84	0.33–2.1	0.707
Time to final assessment (weeks)	1.00	0.99–1.01	0.876	1.00	0.99–1.01	0.692

aOR—adjusted odds ratio; 95% CI—95% confidence interval. ^a^: For both models, the baseline score covariates were collapsed into 3-tier scores, as described in Table 1. Both models met the assumption of proportional odds. ^b^: The aOR is calculated from the log-odds. The calculations were adjusted so that an aOR > 1 indicates higher odds of achieving a superior outcome.

## Data Availability

The data presented in this study are available on request from the corresponding author due to privacy.

## Supplementary Materials

The following supporting information can be downloaded at https://www.mdpi.com/article/10.3390/jcm15176506/s1. File S1: Steroid injection protocol, photographic assessment protocol, and statistical analyses output.

## Abbreviations

The following abbreviations are used in this manuscript:

95% CI	95% confidence interval
aOR	Adjusted odds ratio
LIS	Liquid injectable silicone
