# Supplementary material for: Novel Granulectomy Technique and Intralesional Triamcinolone for Liquid Injectable Silicone-Induced Chin Granulomas: A Long-Term Ambidirectional Cohort Study
Source: J Clin Med. 2026 Aug 22;15(17):6506. doi: 10.3390/jcm15176506 (PMC13566716; doi:10.3390/jcm15176506)
Supplement: Supplementary file 1 [file jcm-15-06506-s001.zip › jcm-4473680-supplementary.pdf]

Postoperative Steroid Injection Protocol

1. The injections are initiated two weeks post-granulectomy, immediately following the removal of subcuticular sutures.
2. The lesions are marked and measured prior to injection.
3. Triamcinolone acetonide (10 mg/mL) suspension is prepared to be administered at 0.1–0.2 mL/cm^2 across the lesion(s).
4. Intralesional injections utilizing a fine-gauge needle (30G) targeted directly into residual fibrous or inflammatory nodules.
5. The injections are administered at 1- to 2-week intervals depending on individual clinical response (i.e. reduction of induration and erythema).
6. A minimum of 3 to a maximum of 7 total injections is targeted. The injections may be titrated to clinical response (e.g. complete resolution of induration or occurrence of flares).

Photographic Assessment

1. Standardized clinical photographs were captured across three angles: anterior, bilateral side profiles, and bilateral obliques.
2. The photographs are graded using the following scale:

Table S1. Evaluation metrics for chin aesthetic and erythema (adapted from Global Aesthetic Improvement Scale)

Scale	5-tier Scale	Description	3-tier scale	Description
Chin aesthetic	5	Very good	4-5	Good
	4	Good		
	3	Fair	3	Fair
	2	Poor	1-2	Poor
	1	Very poor		
Erythema severity	4	Very red	3	Red
	3	Red		
	2	Slightly red	2	Slightly red
	1	Not red at all	1	Not red at all

3. Scoring Workflow
   - Pre-operative and immediate post-operative photographs were graded exclusively by an independent, blinded physician evaluator.
   - Final follow-up aesthetic grades represented the mathematical average of the patient's subjective self-assessment and the blinded physician's objective evaluation.
   - In instances where the average resulted in a fractional decimal (e.g. 3.5), a senior physician acted as a blinded tiebreaker to adjudicate the final integer.

Descriptive Analyses

Overall (N=35)

Case Processing Summary

	Cases					
	Valid		Missing		Total	
	N	Percent	N	Percent	N	Percent
Age	35	100.0%	0	0.0%	35	100.0%
Before_Shape	35	100.0%	0	0.0%	35	100.0%
Before_Red	35	100.0%	0	0.0%	35	100.0%
After_Shape	35	100.0%	0	0.0%	35	100.0%
After_Red	35	100.0%	0	0.0%	35	100.0%

Descriptives

			Statistic	Std. Error
Age	Mean		49.94	1.670
	95% Confidence Interval for Mean	Lower Bound	46.55	
		Upper Bound	53.34	
	5% Trimmed Mean		49.90	
	Median		51.00	
	Variance		97.644	
	Std. Deviation		9.881	
	Minimum		30	
	Maximum		72	
	Range		42	
	Interquartile Range		12	
	Skewness		-.160	.398
	Kurtosis		.276	.778
Before_Shape	Mean		3.3143	.17762
	95% Confidence Interval for Mean	Lower Bound	2.9533	
		Upper Bound	3.6753	
	5% Trimmed Mean		3.4048	
	Median		4.0000	
	Variance		1.104	
	Std. Deviation		1.05081	
	Minimum		1.00	
	Maximum		4.00	
	Range		3.00	

	Interquartile Range		2.00	
	Skewness		-1.008	.398
	Kurtosis		-.707	.778
Before_Red	Mean		3.4857	.13203
	95% Confidence Interval for Mean	Lower Bound	3.2174	
		Upper Bound	3.7540	
	5% Trimmed Mean		3.5714	
	Median		4.0000	
	Variance		.610	
	Std. Deviation		.78108	
	Minimum		1.00	
	Maximum		4.00	
	Range		3.00	
	Interquartile Range		1.00	
	Skewness		-1.521	.398
	Kurtosis		1.886	.778
After_Shape	Mean		4.1429	.14286
	95% Confidence Interval for Mean	Lower Bound	3.8525	
		Upper Bound	4.4332	
	5% Trimmed Mean		4.2143	
	Median		4.0000	
	Variance		.714	
	Std. Deviation		.84515	
	Minimum		2.00	
	Maximum		5.00	
	Range		3.00	
	Interquartile Range		1.00	
	Skewness		-.905	.398
	Kurtosis		.569	.778
After_Red	Mean		2.5429	.17558
	95% Confidence Interval for Mean	Lower Bound	2.1860	
		Upper Bound	2.8997	
	5% Trimmed Mean		2.5476	
	Median		2.0000	
	Variance		1.079	
	Std. Deviation		1.03875	
	Minimum		1.00	
	Maximum		4.00	
	Range		3.00	
	Interquartile Range		1.00	

	Skewness	.046	.398
	Kurtosis	-1.129	.778

Tests of Normality

	Kolmogorov-Smirnov[a]			Shapiro-Wilk		
	Statistic	df	Sig.	Statistic	df	Sig.
Age	.131	35	.136	.957	35	.190
Before_Shape	.429	35	.000	.637	35	.000
Before_Red	.373	35	.000	.690	35	.000
After_Shape	.261	35	.000	.809	35	.000
After_Red	.214	35	.000	.874	35	.001

a. Lilliefors Significance Correction

Steroid Cohort (n=17)

Case Processing Summary[a]

	Cases					
	Valid		Missing		Total	
	N	Percent	N	Percent	N	Percent
Age	17	100.0%	0	0.0%	17	100.0%
Before_Shape	17	100.0%	0	0.0%	17	100.0%
Before_Red	17	100.0%	0	0.0%	17	100.0%
After_Shape	17	100.0%	0	0.0%	17	100.0%
After_Red	17	100.0%	0	0.0%	17	100.0%

a. Injection = Received steroid injection

Descriptives[a]

			Statistic	Std. Error
Age	Mean		51.41	1.908
	95% Confidence Interval for Mean	Lower Bound	47.37	
		Upper Bound	55.46	
	5% Trimmed Mean		51.62	
	Median		53.00	
	Variance		61.882	
	Std. Deviation		7.867	
	Minimum		32	
	Maximum		67	
	Range		35	
	Interquartile Range		8	
	Skewness		-.624	.550
	Kurtosis		1.695	1.063
Before_Shape	Mean		3.3529	.25641
	95% Confidence Interval for Mean	Lower Bound	2.8094	
		Upper Bound	3.8965	
	5% Trimmed Mean		3.4477	
	Median		4.0000	
	Variance		1.118	
	Std. Deviation		1.05719	
	Minimum		1.00	
	Maximum		4.00	
	Range		3.00	
	Interquartile Range		2.00	

	Skewness		-1.180	.550
	Kurtosis		-.292	1.063
Before_Red	Mean		3.5294	.19398
	95% Confidence Interval for Mean	Lower Bound	3.1182	
		Upper Bound	3.9406	
	5% Trimmed Mean		3.6438	
	Median		4.0000	
	Variance		.640	
	Std. Deviation		.79982	
	Minimum		1.00	
	Maximum		4.00	
	Range		3.00	
	Interquartile Range		1.00	
	Skewness		-2.184	.550
	Kurtosis		5.660	1.063
After_Shape	Mean		4.2941	.18718
	95% Confidence Interval for Mean	Lower Bound	3.8973	
		Upper Bound	4.6909	
	5% Trimmed Mean		4.3268	
	Median		4.0000	
	Variance		.596	
	Std. Deviation		.77174	
	Minimum		3.00	
	Maximum		5.00	
	Range		2.00	
	Interquartile Range		1.00	
	Skewness		-.592	.550
	Kurtosis		-.975	1.063
After_Red	Mean		2.5882	.24343
	95% Confidence Interval for Mean	Lower Bound	2.0722	
		Upper Bound	3.1043	
	5% Trimmed Mean		2.5980	
	Median		2.0000	
	Variance		1.007	
	Std. Deviation		1.00367	
	Minimum		1.00	
	Maximum		4.00	
	Range		3.00	
	Interquartile Range		1.50	
	Skewness		.147	.550

	Kurtosis	-1.003	1.063

a. Injection = Received steroid injection

Tests of Normality[a]

	Kolmogorov-Smirnov[b]			Shapiro-Wilk		
	Statistic	df	Sig.	Statistic	df	Sig.
Age	.193	17	.091	.946	17	.392
Before_Shape	.436	17	.000	.629	17	.000
Before_Red	.369	17	.000	.627	17	.000
After_Shape	.290	17	.000	.780	17	.001
After_Red	.251	17	.006	.872	17	.024

a. Injection = Received steroid injection

b. Lilliefors Significance Correction

Control Cohort (n=18)

Case Processing Summary[a]

	Cases					
	Valid		Missing		Total	
	N	Percent	N	Percent	N	Percent
Age	18	100.0%	0	0.0%	18	100.0%
Before_Shape	18	100.0%	0	0.0%	18	100.0%
Before_Red	18	100.0%	0	0.0%	18	100.0%
After_Shape	18	100.0%	0	0.0%	18	100.0%
After_Red	18	100.0%	0	0.0%	18	100.0%

a. Injection = Did not receive steroid injection

Descriptives[a]

			Statistic	Std. Error
Age	Mean		48.56	2.717
	95% Confidence Interval for Mean	Lower Bound	42.82	
		Upper Bound	54.29	
	5% Trimmed Mean		48.28	
	Median		49.50	
	Variance		132.850	
	Std. Deviation		11.526	
	Minimum		30	
	Maximum		72	
	Range		42	
	Interquartile Range		14	
	Skewness		.190	.536
	Kurtosis		-.038	1.038
Before_Shape	Mean		3.2778	.25316
	95% Confidence Interval for Mean	Lower Bound	2.7437	
		Upper Bound	3.8119	
	5% Trimmed Mean		3.3642	
	Median		4.0000	
	Variance		1.154	
	Std. Deviation		1.07406	
	Minimum		1.00	
	Maximum		4.00	
	Range		3.00	
	Interquartile Range		2.00	

	Skewness		-.947	.536
	Kurtosis		-.872	1.038
Before_Red	Mean		3.4444	.18475
	95% Confidence Interval for Mean	Lower Bound	3.0547	
		Upper Bound	3.8342	
	5% Trimmed Mean		3.4938	
	Median		4.0000	
	Variance		.614	
	Std. Deviation		.78382	
	Minimum		2.00	
	Maximum		4.00	
	Range		2.00	
	Interquartile Range		1.00	
	Skewness		-1.031	.536
	Kurtosis		-.445	1.038
After_Shape	Mean		4.0000	.21390
	95% Confidence Interval for Mean	Lower Bound	3.5487	
		Upper Bound	4.4513	
	5% Trimmed Mean		4.0556	
	Median		4.0000	
	Variance		.824	
	Std. Deviation		.90749	
	Minimum		2.00	
	Maximum		5.00	
	Range		3.00	
	Interquartile Range		1.00	
	Skewness		-1.063	.536
	Kurtosis		1.084	1.038
After_Red	Mean		2.5000	.25883
	95% Confidence Interval for Mean	Lower Bound	1.9539	
		Upper Bound	3.0461	
	5% Trimmed Mean		2.5000	
	Median		2.5000	
	Variance		1.206	
	Std. Deviation		1.09813	
	Minimum		1.00	
	Maximum		4.00	
	Range		3.00	
	Interquartile Range		1.50	
	Skewness		.000	.536

	Kurtosis	-1.242	1.038

a. Injection = Did not receive steroid injection

Tests of Normality[a]

	Kolmogorov-Smirnov[b]			Shapiro-Wilk		
	Statistic	df	Sig.	Statistic	df	Sig.
Age	.124	18	.200*	.958	18	.555
Before_Shape	.416	18	.000	.657	18	.000
Before_Red	.372	18	.000	.699	18	.000
After_Shape	.333	18	.000	.784	18	.001
After_Red	.176	18	.147	.878	18	.024

*. This is a lower bound of the true significance.

a. Injection = Did not receive steroid injection

b. Lilliefors Significance Correction

Baseline comparisons—Numeric Data

Group Statistics

	Injection	N	Mean	Std. Deviation	Std. Error Mean
Age	Did not receive steroid injection	18	48.56	11.526	2.717
	Received steroid injection	17	51.41	7.867	1.908

Independent Samples Test

		Levene's Test for Equality of Variances		t-test for Equality of Means						
									95% Confidence Interval of the Difference	
		F	Sig.	t	df	Sig. (2-tailed)	Mean Difference	Std. Error Difference	Lower	Upper
Age	Equal variances assumed	1.969	.170	-.851	33	.401	-2.856	3.356	-9.683	3.971
	Equal variances not assumed			-.860	30.120	.396	-2.856	3.320	-9.635	3.922

Mann-Whitney Test

Ranks

	Injection	N	Mean Rank	Sum of Ranks
Before_Shape	Did not receive steroid injection	18	17.69	318.50
	Received steroid injection	17	18.32	311.50
	Total	35		
After_Shape	Did not receive steroid injection	18	16.53	297.50
	Received steroid injection	17	19.56	332.50
	Total	35		
Before_Red	Did not receive steroid injection	18	17.47	314.50
	Received steroid injection	17	18.56	315.50
	Total	35		
After_Red	Did not receive steroid injection	18	17.64	317.50
	Received steroid injection	17	18.38	312.50
	Total	35		

Test Statistics[a]

	Before_Shape	After_Shape	Before_Red	After_Red
Mann-Whitney U	147.500	126.500	143.500	146.500
Wilcoxon W	318.500	297.500	314.500	317.500
Z	-.223	-.947	-.366	-.223
Asymp. Sig. (2-tailed)	.823	.343	.714	.824
Exact Sig. [2*(1-tailed Sig.)]	.858[b]	.386[b]	.757[b]	.832[b]
Exact Sig. (2-tailed)	.950	.354	.770	.844
Exact Sig. (1-tailed)	.496	.174	.375	.437
Point Probability	.150	.009	.020	.023

a. Grouping Variable: Injection

b. Not corrected for ties.

Baseline comparisons—Categoric Data

Case Processing Summary

	Cases					
	Valid		Missing		Total	
	N	Percent	N	Percent	N	Percent
Before_Shape * Injection	35	100.0%	0	0.0%	35	100.0%
Before_Red * Injection	35	100.0%	0	0.0%	35	100.0%
After_Shape * Injection	35	100.0%	0	0.0%	35	100.0%
After_Red * Injection	35	100.0%	0	0.0%	35	100.0%

Before_Shape * Injection

Crosstab

			Injection		
			Did not receive steroid injection	Received steroid injection	Total
Before_Shape	Very poor	Count	1	1	2
		% within Injection	5.6%	5.9%	5.7%
	Poor	Count	5	4	9
		% within Injection	27.8%	23.5%	25.7%
	Good	Count	12	12	24
		% within Injection	66.7%	70.6%	68.6%
Total		Count	18	17	35
		% within Injection	100.0%	100.0%	100.0%

Chi-Square Tests

	Value	df	Asymptotic Significance (2-sided)	Exact Sig. (2-sided)	Exact Sig. (1-sided)	Point Probability
Pearson Chi-Square	.083[a]	2	.960	1.000		
Likelihood Ratio	.083	2	.959	1.000		
Fisher-Freeman-Halton Exact Test	.377			1.000		
Linear-by-Linear Association	.045[b]	1	.832	.904	.496	.150
N of Valid Cases	35					

a. 4 cells (66.7%) have expected count less than 5. The minimum expected count is .97.

b. The standardized statistic is .211.

Before_Red * Injection

Crosstab

			Injection		
			Did not receive steroid injection	Received steroid injection	Total
Before_Red	Not red at all	Count	0	1	1
		% within Injection	0.0%	5.9%	2.9%
	Slightly red	Count	3	0	3
		% within Injection	16.7%	0.0%	8.6%
	Red	Count	4	5	9
		% within Injection	22.2%	29.4%	25.7%
	Very red	Count	11	11	22
		% within Injection	61.1%	64.7%	62.9%
Total		Count	18	17	35
		% within Injection	100.0%	100.0%	100.0%

Chi-Square Tests

	Value	df	Asymptotic Significance (2-sided)	Exact Sig. (2-sided)	Exact Sig. (1-sided)	Point Probability
Pearson Chi-Square	4.086[a]	3	.252	.319		
Likelihood Ratio	5.628	3	.131	.268		
Fisher-Freeman-Halton Exact Test	3.682			.337		
Linear-by-Linear Association	.103[b]	1	.748	.835	.461	.159
N of Valid Cases	35					

a. 6 cells (75.0%) have expected count less than 5. The minimum expected count is .49.

b. The standardized statistic is .322.

After_Shape * Injection

Crosstab

			Injection		
			Did not receive steroid injection	Received steroid injection	Total
After_Shape	Buruk	Count	2	0	2
		% within Injection	11.1%	0.0%	5.7%
	Tidak ada perubahan	Count	1	3	4
		% within Injection	5.6%	17.6%	11.4%
	Baik	Count	10	6	16
		% within Injection	55.6%	35.3%	45.7%
	Sangat baik	Count	5	8	13
		% within Injection	27.8%	47.1%	37.1%
Total		Count	18	17	35
		% within Injection	100.0%	100.0%	100.0%

Chi-Square Tests

	Value	df	Asymptotic Significance (2-sided)	Exact Sig. (2-sided)	Exact Sig. (1-sided)	Point Probability
Pearson Chi-Square	4.668[a]	3	.198	.218		
Likelihood Ratio	5.500	3	.139	.221		
Fisher-Freeman-Halton Exact Test	4.188			.234		
Linear-by-Linear Association	1.059[b]	1	.303	.330	.207	.096
N of Valid Cases	35					

a. 4 cells (50.0%) have expected count less than 5. The minimum expected count is .97.

b. The standardized statistic is 1.029.

After_Red * Injection

Crosstab

			Injection		
			Did not receive steroid injection	Received steroid injection	Total
After_Red	Tidak merah sama sekali	Count	4	2	6
		% within Injection	22.2%	11.8%	17.1%
	Sedikit merah	Count	5	7	12
		% within Injection	27.8%	41.2%	34.3%
	Merah	Count	5	4	9
		% within Injection	27.8%	23.5%	25.7%
	Sangat merah	Count	4	4	8
		% within Injection	22.2%	23.5%	22.9%
Total		Count	18	17	35
		% within Injection	100.0%	100.0%	100.0%

Chi-Square Tests

	Value	df	Asymptotic Significance (2-sided)	Exact Sig. (2-sided)	Exact Sig. (1-sided)	Point Probability
Pearson Chi-Square	1.083[a]	3	.781	.828		
Likelihood Ratio	1.097	3	.778	.828		
Fisher-Freeman-Halton Exact Test	1.186			.828		
Linear-by-Linear Association	.063[b]	1	.802	.872	.465	.124
N of Valid Cases	35					

a. 6 cells (75.0%) have expected count less than 5. The minimum expected count is 2.91.

b. The standardized statistic is .251.

Final Overall Cohort (n=28)

Case Processing Summary

	Cases					
	Valid		Missing		Total	
	N	Percent	N	Percent	N	Percent
Age	28	100.0%	0	0.0%	28	100.0%
Shape_BeforeFolup	28	100.0%	0	0.0%	28	100.0%
Red_BeforeFolup	28	100.0%	0	0.0%	28	100.0%
Shape_AfterFolup	28	100.0%	0	0.0%	28	100.0%
Red_AfterFolup	28	100.0%	0	0.0%	28	100.0%

Descriptives

			Statistic	Std. Error
Age	Mean		49.36	2.033
	95% Confidence Interval for Mean	Lower Bound	45.19	
		Upper Bound	53.53	
	5% Trimmed Mean		49.22	
	Median		50.50	
	Variance		115.720	
	Std. Deviation		10.757	
	Minimum		30	
	Maximum		72	
	Range		42	
	Interquartile Range		12	
	Skewness		-.014	.441
	Kurtosis		-.091	.858
Shape_BeforeFolup	Mean		4.1429	.16031
	95% Confidence Interval for Mean	Lower Bound	3.8139	
		Upper Bound	4.4718	
	5% Trimmed Mean		4.2143	
	Median		4.0000	
	Variance		.720	
	Std. Deviation		.84828	
	Minimum		2.00	
	Maximum		5.00	
	Range		3.00	
	Interquartile Range		1.00	
	Skewness		-1.072	.441
	Kurtosis		1.191	.858

Red_BeforeFolup	Mean		2.5714	.20203
	95% Confidence Interval for Mean	Lower Bound	2.1569	
		Upper Bound	2.9860	
	5% Trimmed Mean		2.5794	
	Median		2.5000	
	Variance		1.143	
	Std. Deviation		1.06904	
	Minimum		1.00	
	Maximum		4.00	
	Range		3.00	
	Interquartile Range		1.75	
	Skewness		-.004	.441
	Kurtosis		-1.209	.858
Shape_AfterFolup	Mean		3.4643	.20238
	95% Confidence Interval for Mean	Lower Bound	3.0490	
		Upper Bound	3.8795	
	5% Trimmed Mean		3.4603	
	Median		3.0000	
	Variance		1.147	
	Std. Deviation		1.07090	
	Minimum		2.00	
	Maximum		5.00	
	Range		3.00	
	Interquartile Range		1.00	
	Skewness		.100	.441
	Kurtosis		-1.185	.858
Red_AfterFolup	Mean		2.0357	.16652
	95% Confidence Interval for Mean	Lower Bound	1.6940	
		Upper Bound	2.3774	
	5% Trimmed Mean		2.0000	
	Median		2.0000	
	Variance		.776	
	Std. Deviation		.88117	
	Minimum		1.00	
	Maximum		4.00	
	Range		3.00	
	Interquartile Range		2.00	
	Skewness		.277	.441
	Kurtosis		-.896	.858

Tests of Normality

	Kolmogorov-Smirnov[a]			Shapiro-Wilk		
	Statistic	df	Sig.	Statistic	df	Sig.
Age	.104	28	.200*	.960	28	.344
Shape_BeforeFolup	.290	28	.000	.786	28	.000
Red_BeforeFolup	.204	28	.004	.871	28	.003
Shape_AfterFolup	.203	28	.004	.873	28	.003
Red_AfterFolup	.202	28	.005	.851	28	.001

*. This is a lower bound of the true significance.

a. Lilliefors Significance Correction

Final Steroid Cohort (n=13)

Case Processing Summary[a]

	Cases					
	Valid		Missing		Total	
	N	Percent	N	Percent	N	Percent
Age	13	100.0%	0	0.0%	13	100.0%
Shape_BeforeFolup	13	100.0%	0	0.0%	13	100.0%
Red_BeforeFolup	13	100.0%	0	0.0%	13	100.0%
Shape_AfterFolup	13	100.0%	0	0.0%	13	100.0%
Red_AfterFolup	13	100.0%	0	0.0%	13	100.0%

a. Injection = Received steroid injection

Descriptives[a]

			Statistic	Std. Error
Age	Mean		52.46	2.830
	95% Confidence Interval for Mean	Lower Bound	46.30	
		Upper Bound	58.63	
	5% Trimmed Mean		52.51	
	Median		52.00	
	Variance		104.103	
	Std. Deviation		10.203	
	Minimum		32	
	Maximum		72	
	Range		40	
	Interquartile Range		10	
	Skewness		.020	.616
	Kurtosis		.996	1.191
Shape_BeforeFolup	Mean		4.3077	.17485
	95% Confidence Interval for Mean	Lower Bound	3.9267	
		Upper Bound	4.6887	
	5% Trimmed Mean		4.3419	
	Median		4.0000	
	Variance		.397	
	Std. Deviation		.63043	
	Minimum		3.00	
	Maximum		5.00	
	Range		2.00	
	Interquartile Range		1.00	
	Skewness		-.307	.616
	Kurtosis		-.317	1.191

Red_BeforeFolup	Mean		2.6154	.26647
	95% Confidence Interval for Mean	Lower Bound	2.0348	
		Upper Bound	3.1960	
	5% Trimmed Mean		2.6282	
	Median		2.0000	
	Variance		.923	
	Std. Deviation		.96077	
	Minimum		1.00	
	Maximum		4.00	
	Range		3.00	
	Interquartile Range		1.50	
	Skewness		.280	.616
	Kurtosis		-.891	1.191
Shape_AfterFolup	Mean		3.8462	.27377
	95% Confidence Interval for Mean	Lower Bound	3.2497	
		Upper Bound	4.4427	
	5% Trimmed Mean		3.8846	
	Median		4.0000	
	Variance		.974	
	Std. Deviation		.98710	
	Minimum		2.00	
	Maximum		5.00	
	Range		3.00	
	Interquartile Range		2.00	
	Skewness		-.262	.616
	Kurtosis		-.912	1.191
Red_AfterFolup	Mean		1.7692	.23077
	95% Confidence Interval for Mean	Lower Bound	1.2664	
		Upper Bound	2.2720	
	5% Trimmed Mean		1.7436	
	Median		2.0000	
	Variance		.692	
	Std. Deviation		.83205	
	Minimum		1.00	
	Maximum		3.00	
	Range		2.00	
	Interquartile Range		1.50	
	Skewness		.498	.616
	Kurtosis		-1.339	1.191

a. Injection = Received steroid injection

Tests of Normality[a]

	Kolmogorov-Smirnov[b]			Shapiro-Wilk		
	Statistic	df	Sig.	Statistic	df	Sig.
Age	.174	13	.200*	.950	13	.592
Shape_BeforeFolup	.303	13	.002	.778	13	.004
Red_BeforeFolup	.278	13	.007	.862	13	.041
Shape_AfterFolup	.189	13	.200*	.879	13	.070
Red_AfterFolup	.284	13	.005	.785	13	.005

*. This is a lower bound of the true significance.

a. Injection = Received steroid injection

b. Lilliefors Significance Correction

Final Control Cohort (n=15)

Case Processing Summary[a]

	Cases					
	Valid		Missing		Total	
	N	Percent	N	Percent	N	Percent
Age	15	100.0%	0	0.0%	15	100.0%
Shape_BeforeFolup	15	100.0%	0	0.0%	15	100.0%
Red_BeforeFolup	15	100.0%	0	0.0%	15	100.0%
Shape_AfterFolup	15	100.0%	0	0.0%	15	100.0%
Red_AfterFolup	15	100.0%	0	0.0%	15	100.0%

a. Injection = Did not receive steroid injection

Descriptives[a]

			Statistic	Std. Error
Age	Mean		46.67	2.796
	95% Confidence Interval for Mean	Lower Bound	40.67	
		Upper Bound	52.66	
	5% Trimmed Mean		46.41	
	Median		48.00	
	Variance		117.238	
	Std. Deviation		10.828	
	Minimum		30	
	Maximum		68	
	Range		38	
	Interquartile Range		21	
	Skewness		.056	.580
	Kurtosis		-.399	1.121
Shape_BeforeFolup	Mean		4.0000	.25820
	95% Confidence Interval for Mean	Lower Bound	3.4462	
		Upper Bound	4.5538	
	5% Trimmed Mean		4.0556	
	Median		4.0000	
	Variance		1.000	
	Std. Deviation		1.00000	
	Minimum		2.00	
	Maximum		5.00	
	Range		3.00	
	Interquartile Range		1.00	
	Skewness		-.989	.580

	Kurtosis		.407	1.121
Red_BeforeFolup	Mean		2.5333	.30654
	95% Confidence Interval for Mean	Lower Bound	1.8759	
		Upper Bound	3.1908	
	5% Trimmed Mean		2.5370	
	Median		3.0000	
	Variance		1.410	
	Std. Deviation		1.18723	
	Minimum		1.00	
	Maximum		4.00	
	Range		3.00	
	Interquartile Range		3.00	
	Skewness		-.091	.580
	Kurtosis		-1.499	1.121
Shape_AfterFolup	Mean		3.1333	.27372
	95% Confidence Interval for Mean	Lower Bound	2.5463	
		Upper Bound	3.7204	
	5% Trimmed Mean		3.0926	
	Median		3.0000	
	Variance		1.124	
	Std. Deviation		1.06010	
	Minimum		2.00	
	Maximum		5.00	
	Range		3.00	
	Interquartile Range		2.00	
	Skewness		.531	.580
	Kurtosis		-.791	1.121
Red_AfterFolup	Mean		2.2667	.22817
	95% Confidence Interval for Mean	Lower Bound	1.7773	
		Upper Bound	2.7561	
	5% Trimmed Mean		2.2407	
	Median		2.0000	
	Variance		.781	
	Std. Deviation		.88372	
	Minimum		1.00	
	Maximum		4.00	
	Range		3.00	
	Interquartile Range		1.00	
	Skewness		.116	.580
	Kurtosis		-.485	1.121

a. Injection = Did not receive steroid injection

Tests of Normality[a]

	Kolmogorov-Smirnov[b]			Shapiro-Wilk		
	Statistic	df	Sig.	Statistic	df	Sig.
Age	.136	15	.200*	.961	15	.707
Shape_BeforeFolup	.300	15	.001	.806	15	.004
Red_BeforeFolup	.186	15	.171	.858	15	.023
Shape_AfterFolup	.217	15	.056	.862	15	.026
Red_AfterFolup	.219	15	.052	.888	15	.063

*. This is a lower bound of the true significance.

a. Injection = Did not receive steroid injection

b. Lilliefors Significance Correction

Table S2

Hypothesis Test Summary

	Null Hypothesis	Test	Sig.[a,b]	Decision
1	The median of differences between Before_Shape and After_Shape equals 0.	Related-Samples Wilcoxon Signed Rank Test	.001	Reject the null hypothesis.

a. The significance level is .050.

b. Asymptotic significance is displayed.

Confidence Interval Summary

Confidence Interval Type	Parameter	Estimate	95.0% Confidence Interval	
			Lower	Upper
Related-Samples Hodges-Lehman Median Difference	Median of the difference between Before_Shape and After_Shape.	1.000	.500	1.500

Related-Samples Wilcoxon Signed Rank Test

Before_Shape, After_Shape

Related-Samples Wilcoxon Signed Rank Test Summary

Total N	35
Test Statistic	260.500
Standard Error	34.105
Standardized Test Statistic	3.240
Asymptotic Sig.(2-sided test)	.001

Hypothesis Test Summary

	Null Hypothesis	Test	Sig.[a,b]	Decision
1	The median of differences between Before_Red and After_Red equals 0.	Related-Samples Wilcoxon Signed Rank Test	.000	Reject the null hypothesis.

a. The significance level is .050.

b. Asymptotic significance is displayed.

Confidence Interval Summary

Confidence Interval Type	Parameter	Estimate	95.0% Confidence Interval	
			Lower	Upper
Related-Samples Hodges-Lehman Median Difference	Median of the difference between Before_Red and After_Red.	-1.000	-1.000	-.500

Related-Samples Wilcoxon Signed Rank Test

Before_Red, After_Red

Related-Samples Wilcoxon Signed Rank Test Summary

Total N	35
Test Statistic	8.000
Standard Error	33.880
Standardized Test Statistic	-4.191
Asymptotic Sig.(2-sided test)	.000

Table S3

Steroid cohort at final follow up (n=13)

Hypothesis Test Summary

	Null Hypothesis	Test	Sig.[a,b]	Decision
1	The median of differences between Shape_BeforeFolup and Shape_AfterFolup equals 0.	Related-Samples Wilcoxon Signed Rank Test	.190	Retain the null hypothesis.

a. The significance level is .050.

b. Asymptotic significance is displayed.

Confidence Interval Summary

Confidence Interval Type	Parameter	Estimate	95.0% Confidence Interval Lower	Upper
Related-Samples Hodges-Lehman Median Difference	Median of the difference between Shape_BeforeFolup and Shape_AfterFolup.	-.500	-1.000	.500

Related-Samples Wilcoxon Signed Rank Test

Shape_BeforeFolup, Shape_AfterFolup

Related-Samples Wilcoxon Signed Rank Test Summary

Total N	13
Test Statistic	12.000
Standard Error	8.016
Standardized Test Statistic	-1.310
Asymptotic Sig.(2-sided test)	.190

Hypothesis Test Summary

	Null Hypothesis	Test	Sig.^{a,b}	Decision
1	The median of differences between Red_BeforeFolup and Red_AfterFolup equals 0.	Related-Samples Wilcoxon Signed Rank Test	.031	Reject the null hypothesis.

a. The significance level is .050.

b. Asymptotic significance is displayed.

Confidence Interval Summary

Confidence Interval Type	Parameter	Estimate	95.0% Confidence Interval Lower	Upper
Related-Samples Hodges-Lehman Median Difference	Median of the difference between Red_BeforeFolup and Red_AfterFolup.	-.500	-1.500	.000

Related-Samples Wilcoxon Signed Rank Test

Red_BeforeFolup, Red_AfterFolup

Related-Samples Wilcoxon Signed Rank Test Summary

Total N	13
Test Statistic	3.000
Standard Error	6.955
Standardized Test Statistic	-2.157
Asymptotic Sig.(2-sided test)	.031

Control (surgery only) cohort at final follow up (n=15)

Hypothesis Test Summary

	Null Hypothesis	Test	Sig.[a,b]	Decision
1	The median of differences between Shape_BeforeFolup and Shape_AfterFolup equals 0.	Related-Samples Wilcoxon Signed Rank Test	.043	Reject the null hypothesis.

a. The significance level is .050.

b. Asymptotic significance is displayed.

Confidence Interval Summary

Confidence Interval Type	Parameter	Estimate	95.0% Confidence Interval	
			Lower	Upper
Related-Samples Hodges-Lehman Median Difference	Median of the difference between Shape_BeforeFolup and Shape_AfterFolup.	-1.000	-1.500	.000

Related-Samples Wilcoxon Signed Rank Test

Shape_BeforeFolup, Shape_AfterFolup

Related-Samples Wilcoxon Signed Rank Test Summary

Total N	15
Test Statistic	8.000
Standard Error	9.650
Standardized Test Statistic	-2.021
Asymptotic Sig.(2-sided test)	.043

Hypothesis Test Summary

	Null Hypothesis	Test	Sig.[a,b]	Decision
1	The median of differences between Red_BeforeFolup and Red_AfterFolup equals 0.	Related-Samples Wilcoxon Signed Rank Test	.547	Retain the null hypothesis.

a. The significance level is .050.

b. Asymptotic significance is displayed.

Confidence Interval Summary

			95.0% Confidence Interval	
Confidence Interval Type	Parameter	Estimate	Lower	Upper
Related-Samples Hodges-Lehman Median Difference	Median of the difference between Red_BeforeFolup and Red_AfterFolup.	-.250	-1.000	.500

Related-Samples Wilcoxon Signed Rank Test

Red_BeforeFolup, Red_AfterFolup

Related-Samples Wilcoxon Signed Rank Test Summary

Total N	15
Test Statistic	31.500
Standard Error	12.445
Standardized Test Statistic	-.603
Asymptotic Sig.(2-sided test)	.547

Table S4

Note: The raw log odds derived from ordinal logistic regression (SPSS 27) was converted to adjusted odds ratio (aOR) using the following formula:

$$aOR = e^{Estimate}$$

The corresponding confidence intervals were calculated in the same manner.

Multivariable analysis for chin shape at follow up

PLUM - Ordinal Regression

Case Processing Summary

		N	Marginal Percentage
Shape_AfterFolupKat	Poor to very poor	6	21.4%
	Fair	9	32.1%
	Good to very good	13	46.4%
Injection	Did not receive steroid injection	15	53.6%
	Received steroid injection	13	46.4%
Valid		28	100.0%
Missing		0	
Total		28	

Model Fitting Information

Model	-2 Log Likelihood	Chi-Square	df	Sig.
Intercept Only	57.477			
Final	54.148	3.329	3	.344

Link function: Logit.

Goodness-of-Fit

	Chi-Square	df	Sig.
Pearson	51.516	49	.376
Deviance	52.762	49	.331

Link function: Logit.

Pseudo R-Square

Cox and Snell	.112
Nagelkerke	.128
McFadden	.057

Link function: Logit.

Parameter Estimates

		Estimate	Std. Error	Wald	df	Sig.	95% Confidence Interval Lower Bound	95% Confidence Interval Upper Bound
Threshold	[Shape_AfterFolupKat = 1,00]	-2.558	2.141	1.429	1	.232	-6.754	1.637
	[Shape_AfterFolupKat = 2,00]	-.975	2.087	.218	1	.641	-5.065	3.116
Location	Weeks	.001	.005	.024	1	.876	-.008	.010
	ShapeBefore_FolupKat	-.189	.651	.084	1	.771	-1.466	1.087
	[Inj=0]	-1.353	.777	3.031	1	.082	-2.877	.170
	[Inj=1]	0[a]	.	.	0	.	.	.

Link function: Logit.

a. This parameter is set to zero because it is redundant.

Test of Parallel Lines[a]

Model	-2 Log Likelihood	Chi-Square	df	Sig.
Null Hypothesis	54.148			
General	51.846	2.301	3	.512

The null hypothesis states that the location parameters (slope coefficients) are the same across response categories.

a. Link function: Logit.

Multivariable analysis for redness at follow up

PLUM - Ordinal Regression

Case Processing Summary

		N	Marginal Percentage
RedAfter_FolupKat	Not red at all	9	32.1%
	Slightly red	10	35.7%
	Red to very red	9	32.1%
Injection	Did not receive steroid injection	15	53.6%
	Received steroid injection	13	46.4%
Valid		28	100.0%
Missing		0	
Total		28	

Model Fitting Information

Model	-2 Log Likelihood	Chi-Square	df	Sig.
Intercept Only	61.452			
Final	59.112	2.340	3	.505

Link function: Logit.

Goodness-of-Fit

	Chi-Square	df	Sig.
Pearson	56.844	51	.267
Deviance	59.112	51	.203

Link function: Logit.

Pseudo R-Square

Cox and Snell	.080
Nagelkerke	.090
McFadden	.038

Link function: Logit.

Parameter Estimates

		Estimate	Std. Error	Wald	df	Sig.	95% Confidence Interval Lower Bound	Upper Bound
Threshold	[RedAfter_FolupKat = 1,00]	-.367	1.373	.071	1	.789	-3.057	2.323
	[RedAfter_FolupKat = 2,00]	1.234	1.393	.785	1	.376	-1.496	3.965
Location	Weeks	.002	.005	.157	1	.692	-.007	.011
	RedBefore_FolupKat	-.176	.469	.141	1	.707	-1.094	.743
	[Inj=0]	1.064	.730	2.121	1	.145	-.368	2.495
	[Inj=1]	0[a]	.	.	0	.	.	.

Link function: Logit.

a. This parameter is set to zero because it is redundant.

Test of Parallel Lines[a]

Model	-2 Log Likelihood	Chi-Square	df	Sig.
Null Hypothesis	59.112			
General	57.251	1.861	3	.602

The null hypothesis states that the location parameters (slope coefficients) are the same across response categories.

a. Link function: Logit.

Survival Analyses

Kaplan Meier

Case Processing Summary

Injection	Total N	N of Events	Censored N	Censored Percent
Did not receive steroid injection	18	8	10	55.6%
Received steroid injection	17	1	16	94.1%
Overall	35	9	26	74.3%

Survival Table

Injection		Time	Status	Cumulative Proportion Surviving at the Time Estimate	Cumulative Proportion Surviving at the Time Std. Error	N of Cumulative Events	N of Remaining Cases
Did not receive steroid injection	1	43.000	Rekuren	.944	.054	1	17
	2	54.000	Rekuren	.889	.074	2	16
	3	65.000	Rekuren	.833	.088	3	15
	4	76.000	Tidak rekuren	.	.	3	14
	5	98.000	Rekuren	.774	.100	4	13
	6	99.000	Tidak rekuren	.	.	4	12
	7	99.000	Tidak rekuren	.	.	4	11
	8	100.000	Tidak rekuren	.	.	4	10
	9	101.000	Rekuren	.696	.116	5	9
	10	106.000	Tidak rekuren	.	.	5	8
	11	130.000	Tidak rekuren	.	.	5	7
	12	135.000	Rekuren	.597	.136	6	6
	13	147.000	Tidak rekuren	.	.	6	5
	14	169.000	Tidak rekuren	.	.	6	4
	15	169.000	Tidak rekuren	.	.	6	3
	16	215.000	Rekuren	.398	.186	7	2
	17	216.000	Rekuren	.199	.169	8	1
	18	385.000	Tidak rekuren	.	.	8	0
Received steroid injection	1	16.000	Tidak rekuren	.	.	0	16
	2	40.714	Tidak rekuren	.	.	0	15
	3	67.000	Tidak rekuren	.	.	0	14
	4	73.000	Tidak rekuren	.	.	0	13
	5	88.000	Tidak rekuren	.	.	0	12
	6	105.000	Tidak rekuren	.	.	0	11
	7	107.000	Tidak rekuren	.	.	0	10

	8	114.000	Tidak rekuren	.	.	0	9
	9	115.000	Tidak rekuren	.	.	0	8
	10	122.000	Tidak rekuren	.	.	0	7
	11	134.000	Tidak rekuren	.	.	0	6
	12	160.000	Tidak rekuren	.	.	0	5
	13	170.000	Rekuren	.800	.179	1	4
	14	214.000	Tidak rekuren	.	.	1	3
	15	272.000	Tidak rekuren	.	.	1	2
	16	307.000	Tidak rekuren	.	.	1	1
	17	313.000	Tidak rekuren	.	.	1	0

Means and Medians for Survival Time

Injection	Mean[a]				Median			
	Estimate	Std. Error	95% Confidence Interval Lower Bound	95% Confidence Interval Upper Bound	Estimate	Std. Error	95% Confidence Interval Lower Bound	95% Confidence Interval Upper Bound
Did not receive steroid injection	198.447	36.573	126.764	270.130	215.000	74.738	68.514	361.486
Received steroid injection	284.400	25.581	234.262	334.538	.	.	.	.
Overall	258.767	32.058	195.933	321.601	216.000	1.353	213.349	218.651

a. Estimation is limited to the largest survival time if it is censored.

Overall Comparisons

	Chi-Square	df	Sig.
Log Rank (Mantel-Cox)	5.851	1	.016

Test of equality of survival distributions for the different levels of Injection.

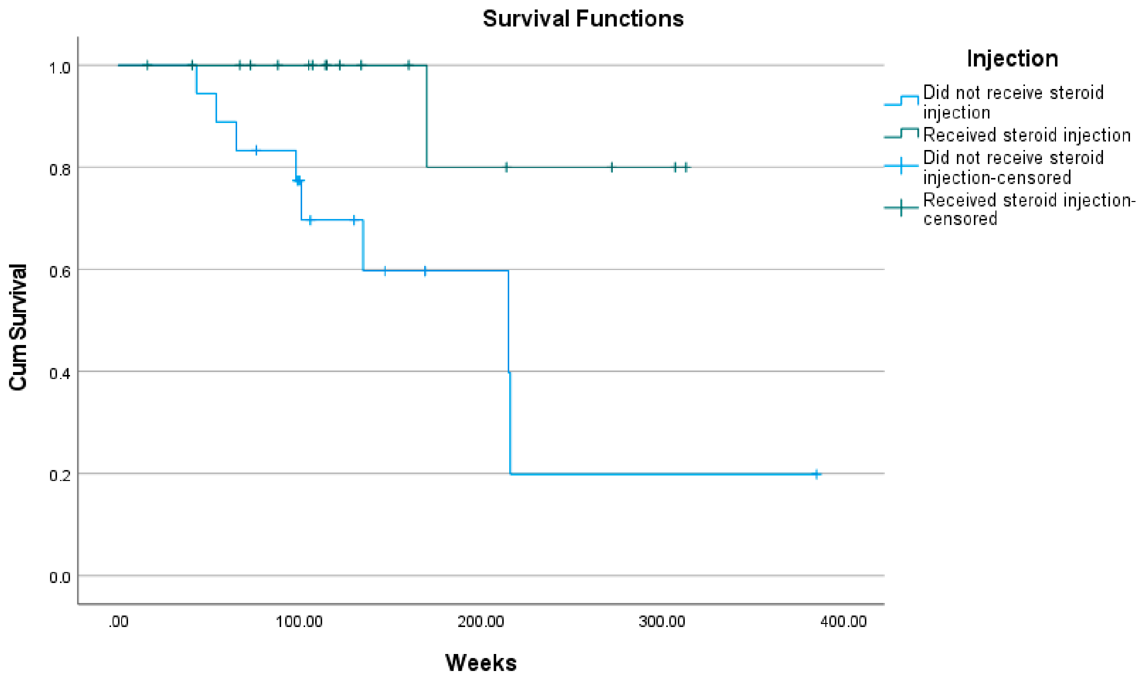

Survival Functions
Injection
Did not receive steroid injection
Received steroid injection
Did not receive steroid injection-censored
Received steroid injection-censored
Cum Survival
1.0
0.8
0.6
0.4
0.2
0.0
.00
100.00
200.00
300.00
400.00
Weeks

Cox Regression

Case Processing Summary

		N	Percent
Cases available in analysis	Event[a]	9	25.7%
	Censored	24	68.6%
	Total	33	94.3%
Cases dropped	Cases with missing values	0	0.0%
	Cases with negative time	0	0.0%
	Censored cases before the earliest event in a stratum	2	5.7%
	Total	2	5.7%
Total		35	100.0%

a. Dependent Variable: Weeks

Categorical Variable Codings[a]

		Frequency	(1)[c]
Injection[b]	0=Did not receive steroid injection	18	1
	1=Received steroid injection	17	0

a. Category variable: Injection (Inj)

b. Indicator Parameter Coding

c. The (0,1) variable has been recoded, so its coefficients will not be the same as for indicator (0,1) coding.

Block 0: Beginning Block

Omnibus Tests of Model Coefficients

-2 Log Likelihood
49.582

Block 1: Method = Enter

Omnibus Tests of Model Coefficients[a]

-2 Log	Overall (score)			Change From Previous Step			Change From Previous Block		
Likelihood	Chi-square	df	Sig.	Chi-square	df	Sig.	Chi-square	df	Sig.
42.976	5.851	1	.016	6.606	1	.010	6.606	1	.010

a. Beginning Block Number 1. Method = Enter

Variables in the Equation

							95,0% CI for Exp(B)	
	B	SE	Wald	df	Sig.	Exp(B)	Lower	Upper
Injection	2.150	1.064	4.081	1	.043	8.585	1.066	69.128

Covariate Means and Pattern Values

	Mean	Pattern	
		1	2
Injection	.545	1.000	.000
